# 4-[5-(4-Fluoro­phen­yl)-1-(4-phenyl-1,3-thia­zol-2-yl)-4,5-dihydro-1*H*-pyrazol-3-yl]-5-methyl-1-(4-methyl­phenyl)-1*H*-1,2,3-triazole

**DOI:** 10.1107/S1600536813008179

**Published:** 2013-03-28

**Authors:** Bakr F. Abdel-Wahab, Seik Weng Ng, Edward R. T. Tiekink

**Affiliations:** a Applied Organic Chemistry Department, National Research Centre, Dokki, 12622 Giza, Egypt; b Department of Chemistry, University of Malaya, 50603 Kuala Lumpur, Malaysia; c Chemistry Department, Faculty of Science, King Abdulaziz University, PO Box 80203 Jeddah, Saudi Arabia

## Abstract

In the title compound, C_28_H_23_FN_6_S, the pyrazole ring adopts an envelope conformation, with the methine C atom being the flap atom. With respect to this ring, the 2-thienyl, triazole and fluoro­benzene rings are approximately coplanar, coplanar and perpendicular, respectively [dihedral angles = 8.56 (17), 6.03 (19) and 73.1 (2)°, respectively] so that to a first approximation the mol­ecule has a T-shape. In the crystal, mol­ecules are consolidated into a three-dimensional architecture by C—H⋯F (involving a bifurcated F atom), C—H⋯S and C—H⋯π inter­actions.

## Related literature

For the synthesis, structure and biological activity of 1-thia­zol-2-ylpyrazoline, see: Abdel-Wahab *et al.* (2012 ▶); Dong *et al.* (2011 ▶).
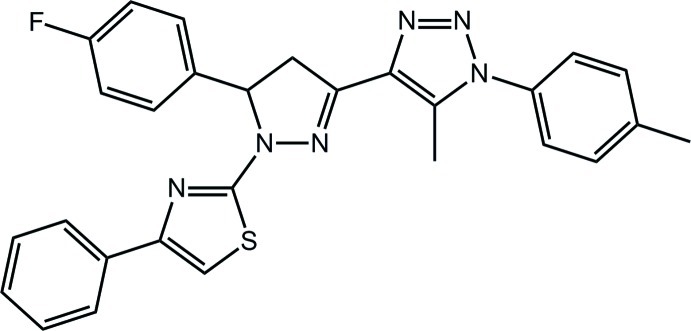


## Experimental

### 

#### Crystal data

C_28_H_23_FN_6_S
*M* *_r_* = 494.58
Monoclinic, 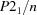

*a* = 17.7373 (18) Å
*b* = 7.8367 (7) Å
*c* = 19.4159 (18) Å
β = 109.323 (11)°
*V* = 2546.8 (4) Å^3^
*Z* = 4
Mo *K*α radiation
μ = 0.16 mm^−1^
*T* = 295 K
0.40 × 0.30 × 0.20 mm

#### Data collection

Agilent SuperNova Dual diffractometer with an Atlas detector
Absorption correction: multi-scan (*CrysAlis PRO*; Agilent, 2011 ▶) *T*_min_ = 0.901, *T*_max_ = 1.000
16284 measured reflections
5871 independent reflections
2694 reflections with *I* > 2σ(*I*)
*R*_int_ = 0.038

#### Refinement

*R*[*F* ^2^ > 2σ(*F* ^2^)] = 0.065
*wR*(*F* ^2^) = 0.232
*S* = 1.06
5871 reflections
328 parameters
H-atom parameters constrained
Δρ_max_ = 0.35 e Å^−3^
Δρ_min_ = −0.38 e Å^−3^

### 

Data collection: *CrysAlis PRO* (Agilent, 2011 ▶); cell refinement: *CrysAlis PRO*; data reduction: *CrysAlis PRO*; program(s) used to solve structure: *SHELXS97* (Sheldrick, 2008 ▶); program(s) used to refine structure: *SHELXL97* (Sheldrick, 2008 ▶); molecular graphics: *ORTEP-3 for Windows* (Farrugia, 2012 ▶) and *DIAMOND* (Brandenburg, 2006 ▶); software used to prepare material for publication: *publCIF* (Westrip, 2010 ▶).

## Supplementary Material

Crystal structure: contains datablock(s) global, I. DOI: 10.1107/S1600536813008179/su2580sup1.cif

Structure factors: contains datablock(s) I. DOI: 10.1107/S1600536813008179/su2580Isup2.hkl

Supplementary material file. DOI: 10.1107/S1600536813008179/su2580Isup3.cml

Additional supplementary materials:  crystallographic information; 3D view; checkCIF report

## Figures and Tables

**Table 1:** Hydrogen-bond geometry (Å, °) *Cg*1 and *Cg*2 are the centroids of the C1–C6 and C22–C27 benzene rings, respectively.

*D*—H⋯*A*	*D*—H	H⋯*A*	*D*⋯*A*	*D*—H⋯*A*
C18—H18⋯S1^i^	0.93	2.87	3.743 (4)	156
C24—H24⋯F1^ii^	0.93	2.55	3.476 (5)	177
C28—H28*B*⋯F1^iii^	0.96	2.53	3.308 (5)	138
C27—H27⋯*Cg*1^i^	0.93	2.75	3.518 (4)	141
C14—H14⋯*Cg*2^ii^	0.93	2.85	3.756 (5)	164

Symmetry codes: (i) 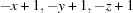; (ii) 
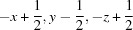
; (iii) 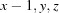.

**Fig. 1.**
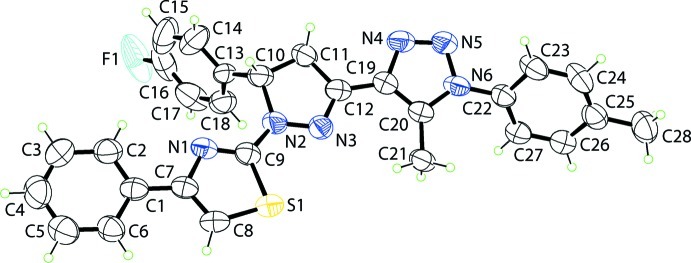
The molecular structure of (I) showing the atom-labelling scheme and displacement ellipsoids at the 35% probability level.

**Fig. 2.**
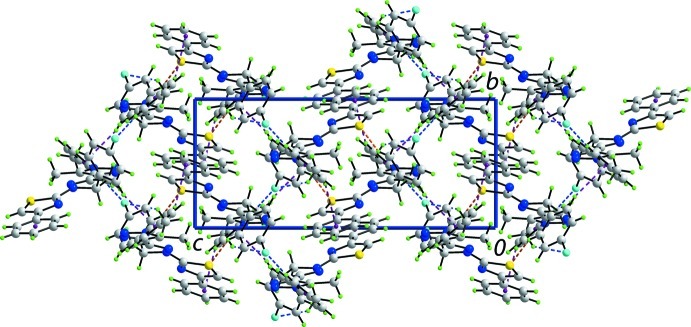
A view of the crystal packing in projection down the a axis. The C—H···F, C—H···S and C—H···π interactions are shown as blue, orange and purple dashed lines, respectively.

## supplementary crystallographic information

### Comment

The title compound, (I), was characterized in relation to on-going studies into the synthesis, structure and biological activities of 1-thiazol-2-ylpyrazoline derivatives (Abdel-Wahab *et al.*, 2012; Dong *et al.*, 2011).

In (I), the pyrazolyl ring adopts an envelope conformation with the methine-C10 atom being the flap atom. The 2-thienyl and triazole rings are approximately co-planar with the central five-membered ring forming dihedral angles of 8.56 (17) and 6.03 (19)°, respectively; the dihedral angle between these rings is 14.33 (17)°, indicating an overall twist in the molecule. By contrast, the fluorobenzene ring is almost perpendicular to the pyrazolyl ring, forming a dihedral angle of 73.1 (2)°. With respect to the respective attached five-membered ring, the phenyl and *p*-tolyl residues form dihedral angles of 2.88 (17) and 45.58 (18)°, respectively, indicating co-planar and inclined dispositions. Overall, the shape of the molecule resembles the distorted T-shape reported for the chlorobenzene derivative (Dong *et al.*, 2011).

The three-dimensional architecture is consolidated by C—H···F, involving a bifurcated F1 atom, C—H···S and C—H···π interactions, Fig. 2 and Table 1.

### Experimental

The title compound was prepared according to the reported method (Abdel-Wahab *et al.*, 2012). Beige crystals were obtained from its DMF solution by slow evaporation at room temperature.

### Refinement

Carbon-bound H-atoms were placed in calculated positions (C—H = 0.93 to 0.98 Å) and were included in the refinement in the riding model approximation, with *U*_iso_(H) = 1.2–1.5*U*_equiv_(C).

### Crystal data

**Table:**

C_28_H_23_FN_6_S	*F*(000) = 1032
*M**_r_* = 494.58	*D*_x_ = 1.290 Mg m^−^^3^
Monoclinic, *P*2_1_/*n*	Mo *K*α radiation, λ = 0.71073 Å
Hall symbol: -P 2yn	Cell parameters from 2615 reflections
*a* = 17.7373 (18) Å	θ = 2.9–27.5°
*b* = 7.8367 (7) Å	µ = 0.16 mm^−^^1^
*c* = 19.4159 (18) Å	*T* = 295 K
β = 109.323 (11)°	Prism, light-brown
*V* = 2546.8 (4) Å^3^	0.40 × 0.30 × 0.20 mm
*Z* = 4	

### Data collection

**Table:**

Agilent SuperNova Dual diffractometer with an Atlas detector	5871 independent reflections
Radiation source: SuperNova (Mo) X-ray Source	2694 reflections with *I* > 2σ(*I*)
Mirror monochromator	*R*_int_ = 0.038
Detector resolution: 10.4041 pixels mm^-1^	θ_max_ = 27.6°, θ_min_ = 2.9°
ω scan	*h* = −23→23
Absorption correction: multi-scan (*CrysAlis PRO*; Agilent, 2011)	*k* = −10→7
*T*_min_ = 0.901, *T*_max_ = 1.000	*l* = −25→25
16284 measured reflections	

### Refinement

**Table:**

Refinement on *F*^2^	Secondary atom site location: difference Fourier map
Least-squares matrix: full	Hydrogen site location: inferred from neighbouring sites
*R*[*F*^2^ > 2σ(*F*^2^)] = 0.065	H-atom parameters constrained
*wR*(*F*^2^) = 0.232	*w* = 1/[σ^2^(*F*_o_^2^) + (0.0967*P*)^2^ + 0.2203*P*] where *P* = (*F*_o_^2^ + 2*F*_c_^2^)/3
*S* = 1.06	(Δ/σ)_max_ = 0.001
5871 reflections	Δρ_max_ = 0.35 e Å^−^^3^
328 parameters	Δρ_min_ = −0.38 e Å^−^^3^
0 restraints	Extinction correction: *SHELXL97* (Sheldrick, 2008), Fc^*^=kFc[1+0.001xFc^2^λ^3^/sin(2θ)]^-1/4^
Primary atom site location: structure-invariant direct methods	Extinction coefficient: 0.0036 (12)

### Special details

**Table:**

Geometry. All e.s.d.'s (except the e.s.d. in the dihedral angle between two l.s. planes) are estimated using the full covariance matrix. The cell e.s.d.'s are taken into account individually in the estimation of e.s.d.'s in distances, angles and torsion angles; correlations between e.s.d.'s in cell parameters are only used when they are defined by crystal symmetry. An approximate (isotropic) treatment of cell e.s.d.'s is used for estimating e.s.d.'s involving l.s. planes.
Refinement. Refinement of *F*^2^ against ALL reflections. The weighted *R*-factor *wR* and goodness of fit *S* are based on *F*^2^, conventional *R*-factors *R* are based on *F*, with *F* set to zero for negative *F*^2^. The threshold expression of *F*^2^ > σ(*F*^2^) is used only for calculating *R*-factors(gt) *etc*. and is not relevant to the choice of reflections for refinement. *R*-factors based on *F*^2^ are statistically about twice as large as those based on *F*, and *R*- factors based on ALL data will be even larger.

### Fractional atomic coordinates and isotropic or equivalent isotropic displacement parameters (Å^2^)

**Table:**

	*x*	*y*	*z*	*U*_iso_*/*U*_eq_	
S1	0.53552 (5)	0.20817 (11)	0.54592 (4)	0.0861 (3)	
F1	0.71777 (15)	0.6920 (6)	0.2659 (2)	0.248 (2)	
N1	0.61200 (15)	0.1843 (3)	0.45456 (13)	0.0749 (7)	
N2	0.48605 (15)	0.3127 (4)	0.40552 (13)	0.0869 (8)	
N3	0.41199 (15)	0.3365 (3)	0.41211 (14)	0.0795 (7)	
N4	0.22891 (18)	0.4311 (4)	0.26442 (14)	0.1003 (9)	
N5	0.15671 (17)	0.4606 (4)	0.26545 (14)	0.1023 (9)	
N6	0.16075 (15)	0.4627 (3)	0.33692 (13)	0.0823 (7)	
C1	0.7421 (2)	0.0649 (4)	0.52615 (17)	0.0782 (8)	
C2	0.7666 (2)	0.0676 (5)	0.4651 (2)	0.0999 (11)	
H2	0.7319	0.1057	0.4205	0.120*	
C3	0.8434 (3)	0.0133 (6)	0.4707 (3)	0.1212 (14)	
H3	0.8594	0.0144	0.4297	0.145*	
C4	0.8952 (3)	−0.0417 (6)	0.5361 (3)	0.1240 (15)	
H4	0.9462	−0.0786	0.5398	0.149*	
C5	0.8711 (3)	−0.0418 (5)	0.5959 (2)	0.1078 (12)	
H5	0.9065	−0.0773	0.6407	0.129*	
C6	0.7966 (2)	0.0089 (4)	0.59155 (19)	0.0888 (9)	
H6	0.7816	0.0061	0.6332	0.107*	
C7	0.66171 (18)	0.1232 (4)	0.52085 (16)	0.0718 (8)	
C8	0.6299 (2)	0.1261 (4)	0.57531 (16)	0.0853 (9)	
H8	0.6561	0.0883	0.6226	0.102*	
C9	0.54482 (19)	0.2344 (4)	0.46099 (16)	0.0738 (8)	
C10	0.48409 (18)	0.3077 (4)	0.32859 (16)	0.0821 (9)	
H10	0.4875	0.1889	0.3141	0.099*	
C11	0.39996 (18)	0.3751 (5)	0.28987 (17)	0.0931 (10)	
H11A	0.4015	0.4900	0.2718	0.112*	
H11B	0.3712	0.3018	0.2494	0.112*	
C12	0.36238 (19)	0.3725 (4)	0.34853 (16)	0.0772 (8)	
C13	0.54996 (17)	0.4088 (4)	0.31593 (15)	0.0739 (8)	
C14	0.5802 (3)	0.3588 (6)	0.2630 (2)	0.1267 (15)	
H14	0.5626	0.2574	0.2379	0.152*	
C15	0.6349 (4)	0.4536 (11)	0.2466 (4)	0.169 (3)	
H15	0.6530	0.4188	0.2090	0.203*	
C16	0.6632 (3)	0.5932 (11)	0.2821 (4)	0.153 (3)	
C17	0.6367 (3)	0.6507 (7)	0.3366 (3)	0.1339 (17)	
H17	0.6568	0.7507	0.3618	0.161*	
C18	0.5788 (2)	0.5554 (5)	0.35322 (19)	0.0985 (11)	
H18	0.5597	0.5918	0.3899	0.118*	
C19	0.27929 (18)	0.4113 (4)	0.33473 (17)	0.0775 (8)	
C20	0.23657 (17)	0.4322 (4)	0.38183 (16)	0.0736 (8)	
C21	0.26213 (18)	0.4199 (4)	0.46230 (16)	0.0849 (9)	
H21A	0.3071	0.3444	0.4793	0.127*	
H21B	0.2189	0.3762	0.4766	0.127*	
H21C	0.2769	0.5310	0.4832	0.127*	
C22	0.08855 (17)	0.4876 (4)	0.35273 (16)	0.0770 (8)	
C23	0.0206 (2)	0.4008 (5)	0.31273 (19)	0.0984 (11)	
H23	0.0212	0.3283	0.2750	0.118*	
C24	−0.0483 (2)	0.4236 (5)	0.3297 (2)	0.1067 (12)	
H24	−0.0941	0.3647	0.3027	0.128*	
C25	−0.0523 (2)	0.5298 (5)	0.3849 (2)	0.0941 (10)	
C26	0.0160 (2)	0.6152 (5)	0.42197 (19)	0.0967 (10)	
H26	0.0154	0.6892	0.4592	0.116*	
C27	0.08582 (19)	0.5965 (4)	0.40657 (17)	0.0853 (9)	
H27	0.1310	0.6581	0.4328	0.102*	
C28	−0.1285 (2)	0.5489 (7)	0.4025 (2)	0.1334 (16)	
H28A	−0.1597	0.4466	0.3889	0.200*	
H28B	−0.1585	0.6439	0.3759	0.200*	
H28C	−0.1159	0.5683	0.4539	0.200*	

### Atomic displacement parameters (Å^2^)

**Table:**

	*U*^11^	*U*^22^	*U*^33^	*U*^12^	*U*^13^	*U*^23^
S1	0.1046 (7)	0.0881 (7)	0.0660 (5)	0.0002 (4)	0.0289 (5)	0.0001 (4)
F1	0.0915 (17)	0.367 (6)	0.276 (4)	0.002 (2)	0.048 (2)	0.218 (4)
N1	0.0815 (16)	0.0787 (17)	0.0566 (14)	−0.0108 (13)	0.0121 (12)	0.0048 (12)
N2	0.0781 (17)	0.118 (2)	0.0608 (15)	−0.0031 (15)	0.0186 (13)	0.0145 (15)
N3	0.0771 (16)	0.0888 (19)	0.0721 (17)	−0.0051 (13)	0.0240 (14)	0.0077 (14)
N4	0.103 (2)	0.128 (3)	0.0606 (17)	0.0176 (18)	0.0145 (15)	−0.0013 (16)
N5	0.090 (2)	0.139 (3)	0.0665 (18)	0.0237 (18)	0.0114 (15)	0.0037 (17)
N6	0.0872 (18)	0.0913 (19)	0.0545 (14)	0.0063 (14)	0.0045 (13)	−0.0023 (13)
C1	0.099 (2)	0.0612 (18)	0.0675 (19)	−0.0033 (16)	0.0189 (18)	−0.0079 (15)
C2	0.110 (3)	0.103 (3)	0.080 (2)	0.010 (2)	0.023 (2)	−0.011 (2)
C3	0.124 (3)	0.138 (4)	0.109 (3)	0.015 (3)	0.049 (3)	−0.027 (3)
C4	0.107 (3)	0.113 (3)	0.145 (4)	0.024 (2)	0.032 (3)	−0.026 (3)
C5	0.116 (3)	0.088 (3)	0.103 (3)	0.014 (2)	0.014 (2)	−0.006 (2)
C6	0.093 (2)	0.080 (2)	0.085 (2)	0.0113 (18)	0.0173 (19)	0.0024 (18)
C7	0.087 (2)	0.0567 (17)	0.0628 (18)	−0.0061 (15)	0.0131 (15)	−0.0022 (14)
C8	0.110 (2)	0.085 (2)	0.0560 (17)	−0.0018 (18)	0.0210 (17)	0.0003 (16)
C9	0.0779 (19)	0.071 (2)	0.0670 (19)	−0.0120 (16)	0.0167 (16)	0.0023 (15)
C10	0.087 (2)	0.093 (2)	0.0625 (18)	−0.0042 (17)	0.0189 (16)	−0.0001 (17)
C11	0.078 (2)	0.125 (3)	0.068 (2)	−0.0075 (19)	0.0123 (16)	0.005 (2)
C12	0.086 (2)	0.080 (2)	0.0606 (18)	−0.0112 (17)	0.0175 (16)	−0.0003 (16)
C13	0.0767 (18)	0.093 (2)	0.0514 (16)	0.0136 (17)	0.0205 (14)	0.0038 (16)
C14	0.161 (4)	0.135 (4)	0.112 (3)	0.027 (3)	0.083 (3)	0.012 (3)
C15	0.143 (5)	0.243 (8)	0.164 (6)	0.060 (5)	0.107 (5)	0.070 (6)
C16	0.069 (2)	0.235 (8)	0.148 (5)	0.006 (4)	0.026 (3)	0.120 (6)
C17	0.118 (3)	0.150 (4)	0.107 (3)	−0.044 (3)	0.001 (3)	0.034 (3)
C18	0.105 (3)	0.113 (3)	0.079 (2)	−0.012 (2)	0.033 (2)	0.000 (2)
C19	0.0764 (19)	0.080 (2)	0.0680 (19)	−0.0005 (15)	0.0126 (16)	0.0001 (16)
C20	0.0786 (19)	0.0703 (19)	0.0626 (18)	−0.0070 (15)	0.0108 (16)	−0.0041 (15)
C21	0.0791 (19)	0.099 (2)	0.0642 (19)	−0.0076 (17)	0.0077 (15)	0.0022 (17)
C22	0.0748 (19)	0.078 (2)	0.0689 (19)	0.0059 (16)	0.0108 (15)	0.0019 (17)
C23	0.086 (2)	0.091 (3)	0.093 (2)	0.0097 (19)	−0.0051 (19)	−0.024 (2)
C24	0.067 (2)	0.099 (3)	0.126 (3)	0.0049 (18)	−0.006 (2)	−0.010 (2)
C25	0.078 (2)	0.104 (3)	0.088 (2)	0.0078 (19)	0.0110 (18)	0.013 (2)
C26	0.088 (2)	0.116 (3)	0.080 (2)	−0.001 (2)	0.0200 (19)	−0.010 (2)
C27	0.085 (2)	0.089 (2)	0.074 (2)	−0.0084 (17)	0.0150 (17)	−0.0117 (18)
C28	0.081 (2)	0.182 (5)	0.130 (3)	0.019 (3)	0.024 (2)	0.031 (3)

### Geometric parameters (Å, º)

**Table:**

S1—C8	1.706 (3)	C11—H11A	0.9700
S1—C9	1.723 (3)	C11—H11B	0.9700
F1—C16	1.355 (6)	C12—C19	1.441 (4)
N1—C9	1.299 (4)	C13—C18	1.364 (5)
N1—C7	1.383 (4)	C13—C14	1.365 (4)
N2—C9	1.371 (4)	C14—C15	1.341 (8)
N2—N3	1.374 (3)	C14—H14	0.9300
N2—C10	1.483 (4)	C15—C16	1.301 (9)
N3—C12	1.288 (4)	C15—H15	0.9300
N4—N5	1.308 (4)	C16—C17	1.369 (8)
N4—C19	1.371 (4)	C17—C18	1.391 (5)
N5—N6	1.366 (3)	C17—H17	0.9300
N6—C20	1.360 (4)	C18—H18	0.9300
N6—C22	1.426 (4)	C19—C20	1.377 (4)
C1—C6	1.388 (4)	C20—C21	1.479 (4)
C1—C2	1.392 (4)	C21—H21A	0.9600
C1—C7	1.468 (4)	C21—H21B	0.9600
C2—C3	1.396 (5)	C21—H21C	0.9600
C2—H2	0.9300	C22—C27	1.363 (4)
C3—C4	1.368 (6)	C22—C23	1.378 (4)
C3—H3	0.9300	C23—C24	1.378 (5)
C4—C5	1.363 (6)	C23—H23	0.9300
C4—H4	0.9300	C24—C25	1.378 (5)
C5—C6	1.356 (5)	C24—H24	0.9300
C5—H5	0.9300	C25—C26	1.362 (5)
C6—H6	0.9300	C25—C28	1.508 (5)
C7—C8	1.353 (4)	C26—C27	1.374 (4)
C8—H8	0.9300	C26—H26	0.9300
C10—C13	1.498 (4)	C27—H27	0.9300
C10—C11	1.526 (4)	C28—H28A	0.9600
C10—H10	0.9800	C28—H28B	0.9600
C11—C12	1.498 (4)	C28—H28C	0.9600
			
C8—S1—C9	88.34 (15)	C18—C13—C10	122.3 (3)
C9—N1—C7	109.7 (2)	C14—C13—C10	119.6 (4)
C9—N2—N3	119.3 (2)	C15—C14—C13	121.0 (5)
C9—N2—C10	122.5 (3)	C15—C14—H14	119.5
N3—N2—C10	113.1 (2)	C13—C14—H14	119.5
C12—N3—N2	108.2 (2)	C16—C15—C14	121.5 (6)
N5—N4—C19	108.9 (2)	C16—C15—H15	119.2
N4—N5—N6	107.2 (2)	C14—C15—H15	119.2
C20—N6—N5	111.0 (2)	C15—C16—F1	122.5 (8)
C20—N6—C22	130.8 (3)	C15—C16—C17	121.0 (5)
N5—N6—C22	118.1 (2)	F1—C16—C17	116.5 (8)
C6—C1—C2	117.7 (3)	C16—C17—C18	118.1 (5)
C6—C1—C7	121.9 (3)	C16—C17—H17	120.9
C2—C1—C7	120.4 (3)	C18—C17—H17	120.9
C1—C2—C3	120.1 (4)	C13—C18—C17	120.3 (4)
C1—C2—H2	120.0	C13—C18—H18	119.8
C3—C2—H2	120.0	C17—C18—H18	119.8
C4—C3—C2	120.4 (4)	N4—C19—C20	109.1 (3)
C4—C3—H3	119.8	N4—C19—C12	119.9 (3)
C2—C3—H3	119.8	C20—C19—C12	131.0 (3)
C5—C4—C3	119.2 (4)	N6—C20—C19	103.9 (3)
C5—C4—H4	120.4	N6—C20—C21	125.5 (3)
C3—C4—H4	120.4	C19—C20—C21	130.6 (3)
C6—C5—C4	121.3 (4)	C20—C21—H21A	109.5
C6—C5—H5	119.3	C20—C21—H21B	109.5
C4—C5—H5	119.3	H21A—C21—H21B	109.5
C5—C6—C1	121.3 (3)	C20—C21—H21C	109.5
C5—C6—H6	119.4	H21A—C21—H21C	109.5
C1—C6—H6	119.4	H21B—C21—H21C	109.5
C8—C7—N1	114.8 (3)	C27—C22—C23	119.7 (3)
C8—C7—C1	126.6 (3)	C27—C22—N6	120.9 (3)
N1—C7—C1	118.6 (3)	C23—C22—N6	119.5 (3)
C7—C8—S1	111.2 (2)	C22—C23—C24	118.7 (3)
C7—C8—H8	124.4	C22—C23—H23	120.6
S1—C8—H8	124.4	C24—C23—H23	120.6
N1—C9—N2	122.9 (3)	C25—C24—C23	122.8 (3)
N1—C9—S1	115.9 (2)	C25—C24—H24	118.6
N2—C9—S1	121.1 (2)	C23—C24—H24	118.6
N2—C10—C13	113.1 (3)	C26—C25—C24	116.3 (3)
N2—C10—C11	100.4 (2)	C26—C25—C28	122.6 (4)
C13—C10—C11	115.1 (3)	C24—C25—C28	121.1 (4)
N2—C10—H10	109.3	C25—C26—C27	122.7 (4)
C13—C10—H10	109.3	C25—C26—H26	118.6
C11—C10—H10	109.3	C27—C26—H26	118.6
C12—C11—C10	103.3 (3)	C22—C27—C26	119.8 (3)
C12—C11—H11A	111.1	C22—C27—H27	120.1
C10—C11—H11A	111.1	C26—C27—H27	120.1
C12—C11—H11B	111.1	C25—C28—H28A	109.5
C10—C11—H11B	111.1	C25—C28—H28B	109.5
H11A—C11—H11B	109.1	H28A—C28—H28B	109.5
N3—C12—C19	123.9 (3)	C25—C28—H28C	109.5
N3—C12—C11	113.4 (3)	H28A—C28—H28C	109.5
C19—C12—C11	122.7 (3)	H28B—C28—H28C	109.5
C18—C13—C14	118.0 (4)		
			
C9—N2—N3—C12	−164.7 (3)	C11—C10—C13—C18	80.7 (4)
C10—N2—N3—C12	−9.1 (4)	N2—C10—C13—C14	149.4 (3)
C19—N4—N5—N6	1.0 (4)	C11—C10—C13—C14	−96.0 (4)
N4—N5—N6—C20	−0.7 (4)	C18—C13—C14—C15	−2.0 (6)
N4—N5—N6—C22	−178.1 (3)	C10—C13—C14—C15	174.9 (4)
C6—C1—C2—C3	0.7 (5)	C13—C14—C15—C16	2.4 (9)
C7—C1—C2—C3	179.5 (3)	C14—C15—C16—F1	−179.2 (4)
C1—C2—C3—C4	−0.5 (6)	C14—C15—C16—C17	−1.5 (10)
C2—C3—C4—C5	−0.4 (7)	C15—C16—C17—C18	0.2 (9)
C3—C4—C5—C6	1.1 (7)	F1—C16—C17—C18	178.1 (4)
C4—C5—C6—C1	−0.8 (6)	C14—C13—C18—C17	0.7 (6)
C2—C1—C6—C5	−0.1 (5)	C10—C13—C18—C17	−176.1 (3)
C7—C1—C6—C5	−178.8 (3)	C16—C17—C18—C13	0.2 (6)
C9—N1—C7—C8	1.2 (4)	N5—N4—C19—C20	−1.0 (4)
C9—N1—C7—C1	−178.0 (2)	N5—N4—C19—C12	178.2 (3)
C6—C1—C7—C8	−2.0 (5)	N3—C12—C19—N4	−173.3 (3)
C2—C1—C7—C8	179.4 (3)	C11—C12—C19—N4	8.5 (5)
C6—C1—C7—N1	177.2 (3)	N3—C12—C19—C20	5.6 (6)
C2—C1—C7—N1	−1.5 (4)	C11—C12—C19—C20	−172.6 (3)
N1—C7—C8—S1	−0.5 (4)	N5—N6—C20—C19	0.1 (4)
C1—C7—C8—S1	178.6 (2)	C22—N6—C20—C19	177.1 (3)
C9—S1—C8—C7	−0.2 (2)	N5—N6—C20—C21	−177.9 (3)
C7—N1—C9—N2	175.1 (3)	C22—N6—C20—C21	−0.9 (5)
C7—N1—C9—S1	−1.3 (3)	N4—C19—C20—N6	0.5 (4)
N3—N2—C9—N1	169.3 (3)	C12—C19—C20—N6	−178.5 (3)
C10—N2—C9—N1	16.1 (5)	N4—C19—C20—C21	178.4 (3)
N3—N2—C9—S1	−14.5 (4)	C12—C19—C20—C21	−0.7 (6)
C10—N2—C9—S1	−167.7 (2)	C20—N6—C22—C27	47.5 (5)
C8—S1—C9—N1	0.9 (2)	N5—N6—C22—C27	−135.7 (3)
C8—S1—C9—N2	−175.6 (3)	C20—N6—C22—C23	−132.9 (4)
C9—N2—C10—C13	−69.1 (4)	N5—N6—C22—C23	44.0 (4)
N3—N2—C10—C13	136.2 (3)	C27—C22—C23—C24	−1.7 (5)
C9—N2—C10—C11	167.7 (3)	N6—C22—C23—C24	178.7 (3)
N3—N2—C10—C11	13.0 (3)	C22—C23—C24—C25	0.1 (6)
N2—C10—C11—C12	−11.4 (3)	C23—C24—C25—C26	1.1 (6)
C13—C10—C11—C12	−133.1 (3)	C23—C24—C25—C28	−179.0 (3)
N2—N3—C12—C19	−177.9 (3)	C24—C25—C26—C27	−0.8 (6)
N2—N3—C12—C11	0.5 (4)	C28—C25—C26—C27	179.2 (3)
C10—C11—C12—N3	7.6 (4)	C23—C22—C27—C26	1.9 (5)
C10—C11—C12—C19	−174.0 (3)	N6—C22—C27—C26	−178.4 (3)
N2—C10—C13—C18	−33.9 (4)	C25—C26—C27—C22	−0.7 (5)

### Hydrogen-bond geometry (Å, º)

Cg1 and Cg2 are the centroids of the C1–C6 and C22–C27 benzene rings, respectively.

**Table:**

*D*—H···*A*	*D*—H	H···*A*	*D*···*A*	*D*—H···*A*
C18—H18···S1^i^	0.93	2.87	3.743 (4)	156
C24—H24···F1^ii^	0.93	2.55	3.476 (5)	177
C28—H28*B*···F1^iii^	0.96	2.53	3.308 (5)	138
C27—H27···*Cg*1^i^	0.93	2.75	3.518 (4)	141
C14—H14···*Cg*2^ii^	0.93	2.85	3.756 (5)	164

Symmetry codes: (i) −*x*+1, −*y*+1, −*z*+1; (ii) −*x*+1/2, *y*−1/2, −*z*+1/2; (iii) *x*−1, *y*, *z*.

## References

[bb1] Abdel-Wahab, B. F., Abdel-Latif, E., Mohamed, H. A. & Awad, G. E. A. (2012). *Eur. J. Med. Chem.* **52**, 263–268. 10.1016/j.ejmech.2012.03.023 22480494

[bb2] Agilent (2011). *CrysAlis PRO* Agilent Technologies, Yarnton, England.

[bb3] Brandenburg, K. (2006). *DIAMOND* Crystal Impact GbR, Bonn, Germany.

[bb4] Dong, W.-J., Cui, F.-H., Gao, Z.-L., Li, R.-S., Shen, G.-L. & Dong, H.-S. (2011). *J. Heterocycl. Chem.* **48**, 1154–1160.

[bb5] Farrugia, L. J. (2012). *J. Appl. Cryst.* **45**, 849–854.

[bb6] Sheldrick, G. M. (2008). *Acta Cryst.* A**64**, 112–122. 10.1107/S0108767307043930 18156677

[bb7] Westrip, S. P. (2010). *J. Appl. Cryst.* **43**, 920–925.

